# The molecular biology of pelvi-ureteric junction obstruction

**DOI:** 10.1007/s00467-017-3629-0

**Published:** 2017-03-13

**Authors:** Laura Jackson, Mark Woodward, Richard J. Coward

**Affiliations:** 1 Bristol Renal Group, University of Bristol, Dorothy Hodgkin Building, Whitson Street, Bristol, BS1 3NY UK; 2 Bristol Royal Hospital for Children, Bristol, UK

**Keywords:** Pelvi-ureteric junction obstruction, Aetiology, Molecular biology, Biomarker, Hydronephrosis

## Abstract

Over recent years routine ultrasound scanning has identified increasing numbers of neonates as having hydronephrosis and pelvi-ureteric junction obstruction (PUJO). This patient group presents a diagnostic and management challenge for paediatric nephrologists and urologists. In this review we consider the known molecular mechanisms underpinning PUJO and review the potential of utilising this information to develop novel therapeutics and diagnostic biomarkers to improve the care of children with this disorder.

## Introduction

Antenatally detected hydronephrosis is a major clinical dilemma for paediatric nephrologists and urologists (incidence of 1 in 200) [1]. This condition has become more prevalent in recent years as antenatal scanning has become more sensitive and widely used. Approximately one in seven neonates with antenatally detected hydronephrosis has pelvi-ureteric junction obstruction (PUJO) [2–4], making PUJO one of the most common causes of congenital urinary tract obstruction, with an incidence of one in 1000 to one in 2000 live births [3–5]. Interestingly, males are affected approximately threefold more frequently than females by this condition [4]. The reason for this difference is unknown.

Intrinsic obstruction due to an adynamic stenotic segment at the PUJ is the most common aetiology (75% of cases) [4], with failure of peristalsis producing an incomplete, functional obstruction. Other causes include: crossing vessels (20%), peripelvic fibrosis, abnormal ureteric insertion, fibroepithelial polyps and anatomical variants, such as retrocaval ureter, horseshoe and duplex kidneys [4, 6, 7].

The major challenge for clinicians is deciding which of these children, who are largely asymptomatic, require a pyeloplasty to relieve the obstruction. This is because two-thirds of children with PUJO do not sustain renal damage or need surgery, and their hydronephrosis spontaneously improves [8–10].

Currently, serial ultrasound and invasive isotope studies are performed to guide surgical management of PUJO [4]. However, the ability of these diagnostic modalities to accurately detect obstruction, identify children at risk of functional deterioration and predict the need for surgery is questionable. Additionally, there remains debate regarding the parameters which indicate clinically significant obstruction [9, 11–13].

In general a pyeloplasty is performed for [6]:

- differential renal function deterioration (differential function of <40% or a fall of >10% on serial MAG3 renograms)
- significant hydronephrosis with a renal pelvis anteroposterior diameter of >3 cm on ultrasound scan
- increasing hydronephrosis with an increasing anteroposterior diameter on serial ultrasound scan
- symptomatic children.

Our current understanding of the natural history of PUJO as well as our ability to distinguish which children require surgery is inadequate. Available diagnostic tests cannot accurately discern between children with PUJO that will resolve spontaneously and those with PUJO that will persist, causing functional impairment. Consequently, despite radiological monitoring, there is a risk of loss of function in the affected kidney while the patient is under observation [14].

In this review we discuss the currently known molecular mechanisms underlying intrinsic PUJO and whether this information could contribute to the future development of novel therapies and diagnostic biomarkers.

## Anatomy of the upper urinary tract

**Fig. 1:**
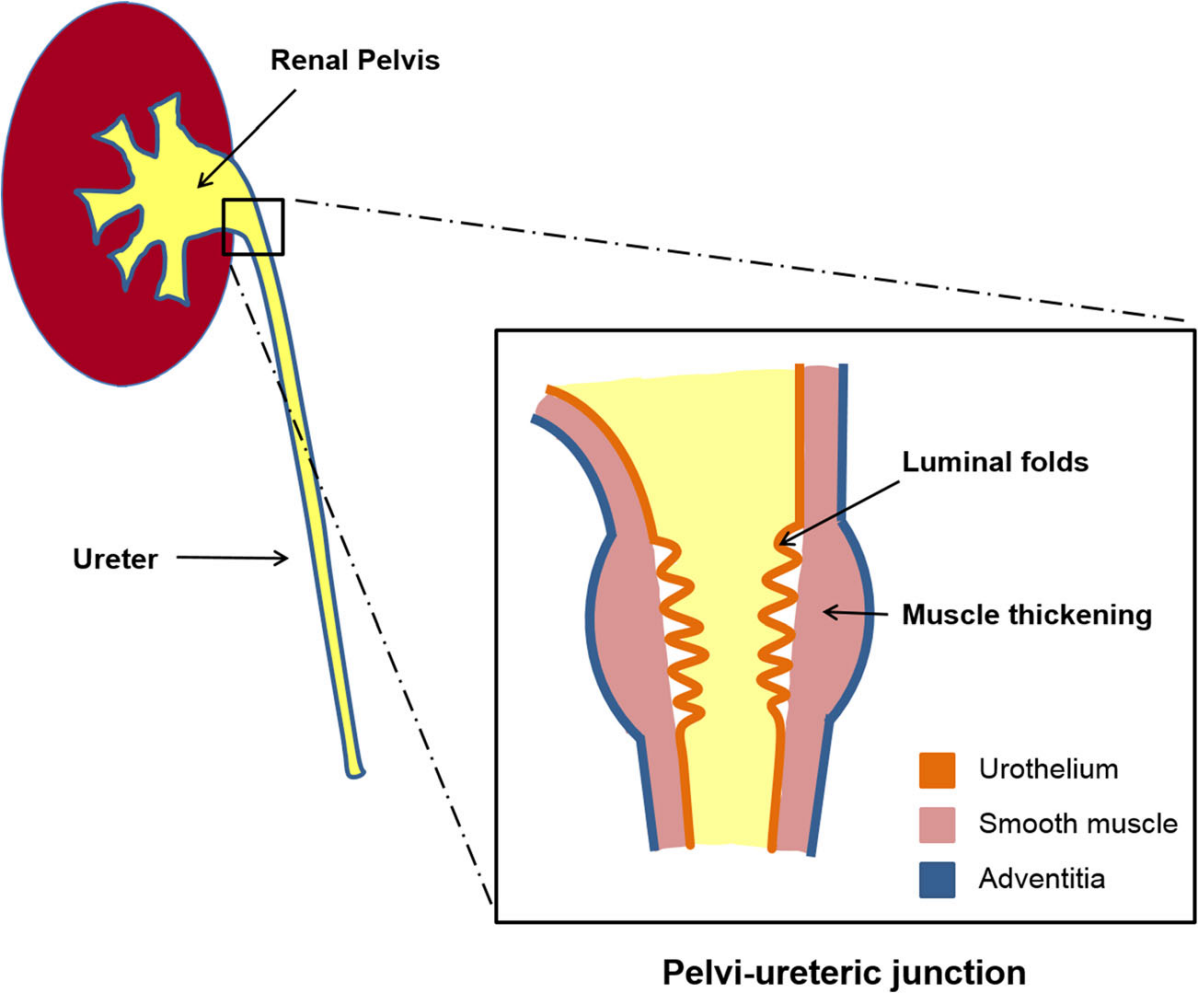
Diagrammatic representation of the pelvi-ureteric junction (PUJ). The gradual transition from the renal pelvis to the proximal ureter is illustrated as well as the increased mucosal folds and smooth muscle thickening in this region

The PUJ is a region of gradual transition from the funnel-shaped renal pelvis to the proximal ureter [15] (Fig. 1). It is a physiologic sphincter [16] that is characterised by prominent luminal folds with increased muscle thickness capable of creating a high-pressure zone to regulate urine flow. Similar to the adjacent renal pelvis and ureter, the PUJ comprises three main layers: the inner urothelium, middle smooth muscle and outer adventitia [15]. Smooth muscle contraction propels urine from the renal pelvis to the bladder [17], coordinated by submucosal and intra-muscular nerve plexi [18] and modulated by autonomic innervation involving a range of neurotransmitters that include acetylcholine, noradrenaline, substance P, neurokinin A, calcitonin gene-related peptide, neuropeptide Y, vasoactive intestinal peptide and nitric oxide (NO) [17].

## Embryology of the ureter and PUJ

**Fig. 2:**
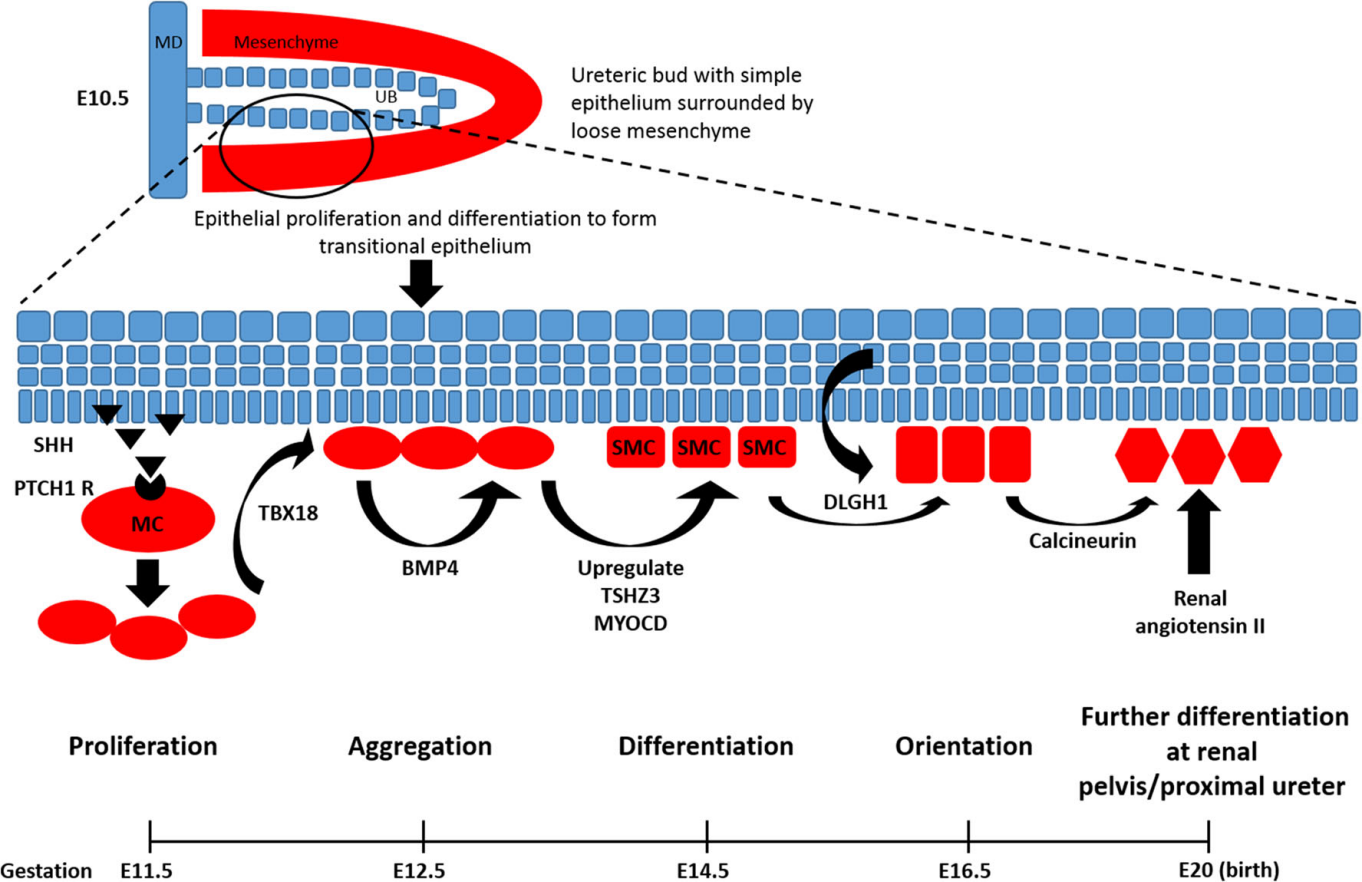
Embryological signalling pathways of the PUJ. The ureteric bud arises from the mesonephric duct and initially consists of only a simple epithelial layer extending into loose mesenchyme. Epithelial cell proliferation and differentiation to form transitional epithelium leads to luminal obliteration, which at the end of the embryonic period is corrected by physiologic recanalisation of the ureter. Epithelial paracrine and mesenchymal autocrine signalling stimulates the proliferation and differentiation of the mesenchyme into smooth muscle cells (*SMC*) which aggregate and orientate so as to encircle the epithelial tube. Specifically, the urothelium secretes SHH which activates the PTCH1 receptor on adjacent mesenchyme, thereby stimulating mesenchymal proliferation. Mesenchymal cells (*MC*) express TBX18, a T-box transcription factor, which enables the correct localisation and aggregation of the former around the urothelium. The mesenchymal cells also express BMP4 which acts in an autocrine manner to upregulate TSHZ3 and MYODC. MYODC enables differentiation of SMC by increasing the transcription of genes encoding smooth muscle contractile proteins. DLGH1, expressed by the urothelium and SMC, is responsible for the correct orientation of SMC around the urothelial tube. In postnatal mice (equivalent to second trimester of gestation in humans), increased urine production matches the development of the renal pelvis and is accompanied by a second phase of muscle differentiation that particularly affects the renal pelvis and proximal ureter, regulated by calcineurin and angiotensin II signalling. The timeline refers to days of gestation (*E* embryonic day) in mouse models.* MD* Mesonephric duct,* UB* ureteric bud,* MC* mesenchymal cells. See Table 1 for description of factors active in the pathways involved in ureteric development

Understanding the normal embryology of PUJ formation is vital when considering where development may proceed incorrectly in congenital abnormalities such as PUJO. The kidney develops from metanephric mesoderm as far along the nephron as the distal tubules. The collecting duct onwards, including the major and minor calyces, renal pelvis and ureter has a different embryological origin, arising from the ureteric bud [19, 20]. Thus, the PUJ does not represent an embryological fusion site, rather it is derived exclusively from the ureteric bud. The important molecular pathways that form the ureter and PUJ are shown in Fig. 2 and Table 1 [15, 26–28]. Briefly, the ureteric bud, consisting of a simple epithelial layer extending into loose mesenchyme, arises from the mesonephric duct during the fifth week of gestation in humans [26]. Epithelial cell proliferation and differentiation then results in the formation of the transitional epithelium. Epithelial paracrine and mesenchymal autocrine signalling stimulates the formation of smooth muscle cells from mesenchyme, which begins at 12 weeks of gestation in humans [26, 29]. Mouse models have implicated a number of signalling molecules in this process of proliferation, aggregation, differentiation and orientation of smooth muscle cells as they encircle the urothelial tube (Fig. 2, Table 1). A second phase of smooth muscle differentiation that particularly affects the renal pelvis and proximal ureter occurs in postnatal mice (equivalent to the second trimester of gestation in humans) and is regulated by calcineurin and angiotensin II signalling [30, 31].

**Table 1:** Proteins/molecular pathways involved in ureteric development

Protein	Full protein name	Function	Reference
SHH	Sonic hedgehog	Morphogen which stimulates peri-urothelial mesenchymal cell proliferation and regulates timing of smooth muscle cell differentiation	[21]
PTCH1 receptor	Protein patched homolog 1	Receptor for SHH, functions as tumour suppressor when unbound	[21]
BMP4	Bone morphogenetic protein 4	Growth factor, necessary for smooth muscle cell differentiation and ureter morphogenesis	[22]
TSHZ3	Teashirt zinc finger homeobox 3	Transcription factor-like protein necessary for myocardin expression and ureteric smooth muscle cell differentiation	[23]
MYOCD	Myocardin	Transcriptional co-activator, necessary for expression of contractile proteins	[23]
TBX18	T Box protein 18	Transcription factor necessary for correct localisation and aggregation of smooth muscle cells around ureteric urothelium	[24]
DLGH1	Disks large homolog 1	Scaffolding protein, regulates smooth muscle cell orientation	[25]

## Pathologic features of intrinsic PUJO

**Fig. 3:**
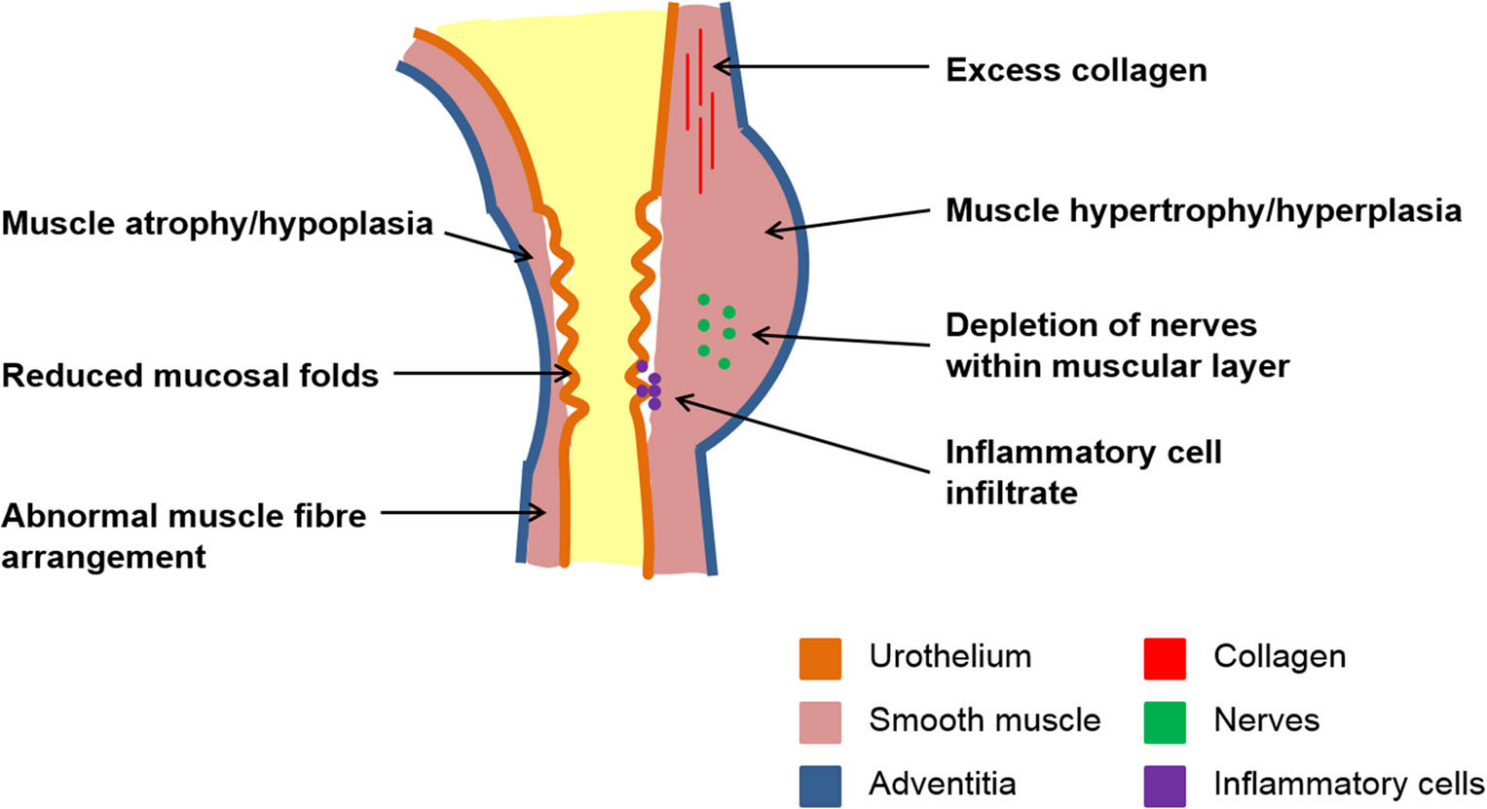
Pathologic features of intrinsic PUJO. Reduced luminal mucosal folds, excess collagen deposition, depletion of nerves within the muscular layer, abnormal muscle fibre arrangement, inflammatory infiltrate and both muscle hypertrophy/hyperplasia and muscle atrophy/hypoplasia are seen at the PUJ in human PUJO

Inflammatory cell infiltration [32], varying degrees of fibrosis, excess collagen deposition [32–35] and abnormal muscle fibre arrangement [36] are present in human intrinsic PUJ obstruction. Both muscular hypertrophy/hyperplasia [32, 34, 37] and atrophy/hypoplasia [32, 36] are reported alongside depletion of nerves to the muscular layer [33]. These findings are noted when the PUJ is excised at pyeloplasty and therefore represent late features of PUJ obstruction (Fig. 3). Although the time course of PUJ disease progression is unknown in humans, genetic mouse models of hydronephrosis show abnormalities of peri-urothelial mesenchymal organisation as early as embryonic day (E) 12.5 (approximately equivalent to 35 days of gestation in humans) [24] and smooth muscle cell differentiation at E15.5 (approximately equivalent to 12 weeks of gestation in humans) [23]. One week postnatally (approximately equivalent to humans at birth) mice with Id2 haploinsufficiency show smooth muscle irregularity and hypertrophy at the PUJ [38], features which are common to human PUJO. The possible mechanisms underlying this pathology are described later in this review.

## Modelling PUJO to understand its molecular biology

**Fig. 4:**
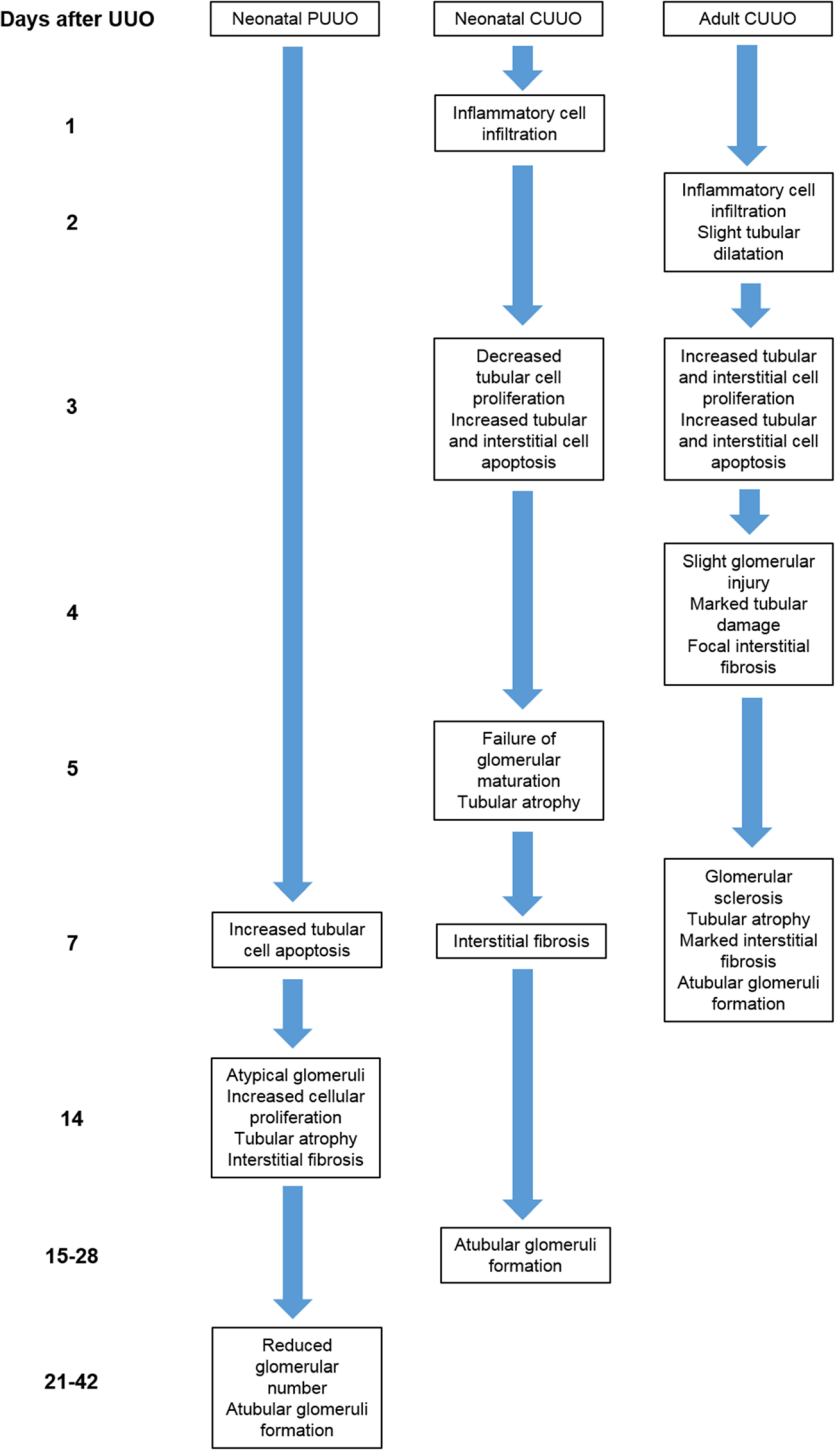
Pathologic features of rodent models of unilateral ureteric obstruction (*UUO*). Timeline of the development of renal pathogenic features in neonatal and adult models of UUO.* CUUO* Complete UUO,* PUUO* partial UUO

Adult and neonatal rodent models of complete and partial unilateral ureteric obstruction (UUO) have been extensively used to investigate the molecular biology of congenital obstructive nephropathy. Neonatal models are particularly helpful because rodent nephrogenesis continues for 1 week postnatally and nephron maturation over the subsequent week. Thus, at birth and 1 week of age, rodent kidney development is equivalent to humans at the second trimester of gestation and birth, respectively [11]. This gives a window in which surgery can be performed on the animals to mimic in utero obstruction in humans. Adult obstructive models show a broadly similar pathologic progression to neonatal models with the exception that neonatal obstruction impedes normal maturation and growth of the kidney and leads to early nephron loss. The renal pathologic findings in neonatal and adult UUO models and the timescale of their development are presented in Fig. 4 [39–47].

A comprehensive review comparing neonatal models with human disease confirms their validity for investigating obstructive nephropathy and will not be further discussed in this review [48].

## Proposed molecular mechanisms underpinning PUJO

In the following subsections we highlight some of the molecular steps that may lead to the development of intrinsic PUJO and subsequent obstructive nephropathy. Data have been obtained from both adult and neonatal models of complete and partial ureteric obstruction alongside evaluation of tissue obtained at pyeloplasty for human PUJ obstruction.

### Neurogenic factors

Light microscopy studies have revealed reduced innervation within the muscular layer of the PUJ in human specimens excised at pyeloplasty for PUJO [33]. This is associated with reduced expression of molecular markers, including glial cell line-derived neurotrophic factor (survival factor for neurons), protein gene product 9.5 (general neuronal marker), and nerve growth factor receptor protein, in the muscle layers of the stenotic PUJ compared to controls. Although it is speculated that these neuronal changes may contribute to the pathogenesis of PUJO, there is as yet no evidence to confirm or refute this notion. Conflicting changes in synaptophysin (e.g. major synaptic vesicle protein p38) expression in terms of both amount (increased and decreased) and distribution (localisation to the nucleus) are reported in PUJO compared to controls and are of uncertain significance. S-100 (schwann cell marker) and neurofilament (neuronal protein) expression are unchanged, demonstrating there is not a global reduction in neuronal components [34, 49].

### Myogenic factors

Together with increased smooth muscle cell apoptosis, phenotypic and cytoskeletal smooth muscle cell changes are seen in the human PUJ excised at pyeloplasty for PUJO. The stenotic PUJ shows significantly increased expression of smooth muscle myosin heavy chain isoforms 1 and 2 [37], as well as an altered ratio of integrin (transmembrane signalling receptor) isoform expression compared to control samples [50]. The preferential expression of immature integrins in the stenotic PUJ [50] may indicate developmental delay of the smooth muscle cells, potentially contributing to their altered function and increased apoptosis in PUJO.

Supporting a myogenic cause of PUJO, transgenic mouse models targeting smooth muscle differentiation generate a PUJ phenotype with hydronephrosis secondary to functional obstruction (Table 2).

**Table 2:** Evidence from animal and human studies of genes potentially involved in the pathogenesis of pelvi-ureteric junction obstruction

Gene	Full gene name	Animal	Features and mechanism	Human	Reference
*Ace*	Angiotensin converting enzyme	*Ace* ^−/−^ mice	Hydronephrosis, renal parenchymal atrophy		[51]
*Adamts-1*	A disintegrin-like and metallopeptidase with thrombospondin type 1 motif, 1	*Adamts* ^-1−/−^ mice	PUJ obstruction, increased collagen at PUJ. Other urogenital anomalies.		[52]
*Agt*	Angiotensin	*Agt* ^−/−^ mice	Hydronephrosis, renal parenchymal atrophy,		[53]
*Agtr 1a/b*	Angiotensin II receptor type 1 (1a and 1b)	*Agtr1* ^−/−^ (*1a* and* 1b*) mice	Hydronephrosis in older mice, renal parenchymal atrophy, failure of renal pelvis development, ureteric smooth muscle hypoplasia and abnormal peristalsis		[31]
*Aqp2*	Aquaporin 2	*Aqp* ^2S256L/S256L^ CPH mice	Mutation in CPH mice prevents Aqp2 phosphorylation and normal trafficking. Hydronephrosis secondary to polyuria		[54]
*Calcineurin*	Calcineurin. Also known as Protein phosphatase 3 (ppp3)	*Pax3-Cre* ^T/+^;* Cnb1* ^flox/ flox^ mice	Calcineurin inactivation in metanephric and ureteral mesenchyme giving hydronephrosis, abnormal pyeloureteral peristalsis with defective renal pelvis and smooth muscle development		[30]
*Id2*	Inhibitor of DNA binding 2	*Id2* ^−/−^ and* Id2* ^ +/−^ mice	Hydronephrosis and PUJ development		[38]
*Nfia*	Nuclear factor I/A	*Nfia* ^+/−^ and* Nfia* ^−/−^ mice	Hydroureteronephrosis, VUR, abnormal PUJ and VUJ development. CNS malformations.	*Nfia * ^+/−^due to chromosomal translocation and deletion. VUR and CNS malformations.	[55]
*TBX18*	T-box transcription factor	*Tbx18* ^−/−^ mice	Hydroureteronephrosis, short ureters, ureteric smooth muscle defects due to abnormal smooth muscle cell differentiation and localisation	Hispanic family with autosomal dominant CAKUT predominantly PUJO. Heterozygous truncating mutation (c.1010delG) of* Tbx18*	[24, 56]
*Tshz2* and* 3*	Teashirt zinc finger family member 2 and 3	*Tshz3* ^−/− ^mice	Hydronephrosis with PUJ configuration, abnormal smooth muscle differentiation proximal ureter	*Tshz2*/*Tshz3* mutations not cause of PUJO in Albanian/Macedonian population	[57, 58]

CAKUT, Congenital anomalies of the kidney and urinary tract; CNS, central nervous system; CPH, congenital progressive hydronephrosis; PUJO, pelvi-ureteric junction obstruction; VUJ, vesico-ureteric junction; VUR, vesico-ureteric reflux

### Increased pressure, impeded blood supply and hypoxia

**Fig. 5:**
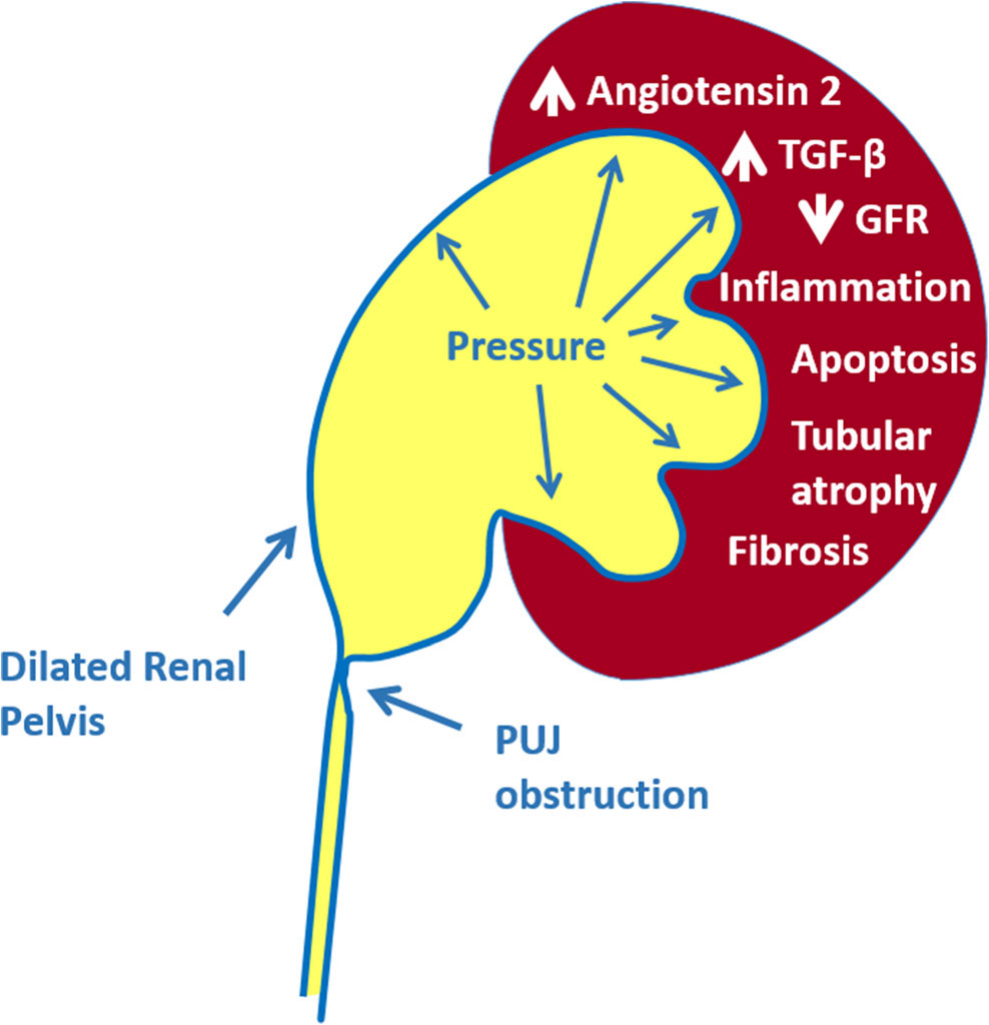
Major mechanisms of renal injury in PUJO.* GFR* glomerular filtration rate,* TGF* transforming growth factor

Obstructive hydronephrosis is associated with a doubling to trebling of renal pelvis pressure [16, 59–61]. The resultant increased intratubular hydrostatic pressure [62] stimulates the renopathogenic effects of obstruction via three proposed mechanisms, namely, (1) tubular ischaemia due to hypoperfusion, (2) pressure-induced mechanical stretch/compression of tubular cells and (3) altered urinary shear stress. The latter two mechanisms are likely to be the primary inducers of obstructive renal injury [48], causing dysregulation of many cytokines, growth factors, enzymes and cytoskeletal proteins (Table 3), resulting in early renal haemodynamic changes followed by structural and functional alterations to the entire nephron. Figure 5 highlights the major mechanisms of renal injury in PUJO.

**Table 3:** Table showing the major cytokines, growth factors, chemokines, enzymes and cytoskeletal proteins which demonstrate altered intra-renal regulation in obstructive nephropathy, the timing of these changes and their mode of action

Protein^a^	Action	Change/timing	Species	Reference
Angiotensin II	Vasoregulatory, proinflammatory, proapoptotic, profibrotic	Increased 28 days Increased 1 week and 5 weeks Increased after mechanical stretch	Neonatal rat CUUO Adult rat CUUO In vitro podocytes	[63] [64] [65]
α-SMA	Increases myofibroblast contractility/EMT marker	Increased 5 days Increased 4 days	Neonatal rat CUUO Adult mouse CUUO	[39] [66]
Caspases	Proapoptotic	Increased 14 days Increased 1 day	Neonatal rat CUUO Adult rat CUUO	[67] [68]
Clusterin	Cytoprotective via pro-survival autophagy	Increased 5 days	Neonatal rat CUUO	[39]
COX-2	Polyuria and natriuresis, anti-apoptotic, antifibrotic	Increased 24 h Increased 3 days (mRNA)	Adult rat CBUO Adult mouse CUUO	[69] [70]
CTGF	Profibrotic	Increased 2 days (mRNA)	Adult rat CUUO	[45]
EGF	Epithelial survival factor	Decreased 7 days (mRNA) (Undetectable expression in neonatal rat kidney before 4 days) Decreased 33 days Decreased at pyeloplasty (mean age 2 years) (mRNA) Decreased at pyeloplasty (mean age 5 years)	Neonatal rat CUUO Neonatal rat both CUUO and 5 day CUUO then release Human renal biopsy Human renal biopsy	[71] [39] [72] [73]
ET-1	Vasoconstrictor	Increased 2 days (mRNA)	Adult rat CUUO	[45]
Fas-L	Proapoptotic	Increased 1 day (mRNA)	Adult rat CUUO	[68]
HSP-70	Antiapoptotic	Decreased 14 days	Neonatal CUUO	[67]
ICAM-1	Proinflammatory	Increased 3 days	Adult mouse CUUO	[74]
Il-6	Proinflammatory	Increased 2 days (mRNA)	Adult rat CUUO	[45]
Integrin (β1)	Profibrotic	Increased 3 days Increased after mechanical stretch	Adult mouse CUUO In vitro proximal tubular cells	[75] [76]
MCP-1	Proinflammatory	Increased 12 days, no change 4 days Increased 2 days (mRNA) Increased at pyeloplasty (mean age 2 years) (mRNA)	Neonatal rat CUUO Adult rat CUUO Human renal biopsy	[77] [45] [72]
MMP 2 and 9	ECM degradation	Decreased 3 days	Adult mouse CUUO	[74]
PAI-1	Profibrotic, inhibits ECM degradation	Increased 7 days	Adult mouse CUUO	[78]
PDGF	Profibrotic	Increased 4 days	Adult mouse CUUO	[66]
NF-κB	Regulatory transcription factor	Increased 2 days	Adult mouse CUUO	[45]
Nitric oxide	Vasodilator, anti-apoptotic, antifibrotic	Decreased 14 days	Neonatal rat CUUO	[67, 79]
Renin	Cleaves angiotensinogen, upregulates renin–angiotensin system	Increased 3 days (mRNA) Increased 5 days Increased 14 days (mRNA) Increased 4–5 weeks Increased 24 h Increased after mechanical stretch	Neonatal rat CUUO Neonatal rat CUUO Neonatal rat CUUO Neonatal rat CUUO Adult rat CUUO In vitro proximal tubular cells	[71] [39] [63] [80] [81] [82]
TGF-β	Proinflammatory, proapoptotic, profibrotic, stimulates EMT	Increased 1 day (mRNA) Increased 33 days Increased 3 days (mRNA) Increased at pyeloplasty (mean age 5 years)	Neonatal rat CUUO Neonatal rat both CUUO and 5 day CUUO then release Adult rat CUUO Human renal biopsy	[71] [39] [83] [73]
TIMP-1	Profibrotic, inhibits ECM degradation	Increased 5 days Increased 3 days	Adult rat CUUO Adult mouse CUUO	[84] [74]
TNF-α	Proapoptotic, proinflammatory	Increased 14 days (mRNA) Increased 1 day Increased 2 days (mRNA) Increased 1 day	Neonatal rat CUUO Adult rat CUUO Adult rat CUUO Adult rat CUUO	[85] [68] [45] [68]
VCAM-1	Proinflammatory	Increased 3 days (mRNA)	Adult mouse CUUO	[86]
VEGF (podocytes)	Endothelial survival factor	Increased 28 days Decreased 14 days	Neonatal PUUO Neonatal CUUO	[87] [87]
VEGF (tubules)	Endothelial survival factor	Variable expression Decreased 14 days	Neonatal PUUO Neonatal CUUO	[87] [87]
Vimentin	Intermediate filament protein/ EMT marker	Increased 5 days	Neonatal rat CUUO	[39]
WT-1	Transcriptional regulator, key role in renal development	Decreased 14 days	Neonatal rat CUUO	[85]

Change is compared to sham animal or control human kidney and refers to protein expression unless otherwise stated. Timing is days after creation of unilateral ureteric obstruction (UUO)

CUUO, Complete UUO; *CBUO* complete bilateral ureteric obstruction; PUUO, partial UUO

^a^α-SMA, Alpha-smooth muscle actin; COX-2, cyclooxygenase 2; CTGF, connective tissue growth factor; ECM, extracellular matrix; EGF, epidermal growth factor; EMT, epithelial–mesenchymal transition; FasL, Fas ligand; HSP-70, heat shock protein 70; ICAM-1, intercellular adhesion molecule 1; IL-6, interleukin-6; MCP-1, monocyte chemoattractant protein 1; MMP, matrix metalloproteinase; NF-κB, nuclear factor kappa-light-chain-enhancer of activated B cells; TGF-β, transforming growth factor-beta; TIMP-1, tissue inhibitor of metalloproteinases 1; TNF-α , tumour necrosis factor-alpha; VCAM-1, vascular cell adhesion molecule 1; VEGF, vascular endothelial growth factor; WT-1, Wilms tumor protein

Following a short initial increase in renal blood flow related to local vasodilator production [48], the intra-renal renin–angiotensin–aldosterone system (RAAS) is activated causing pre- and post-glomerular vasoconstriction and a resultant fall in renal blood flow (RBF), medullary oxygen tension and glomerular filtration rate (GFR) [11, 48, 64, 80, 88–90]. Proximal tubular hypoxia and necrosis in neonatal rats with UUO suggest that vasoconstriction causes segment-specific ischaemic injury [91]. Accordingly, angiotensin II receptor, type 1 (AT1 receptor) inhibition improves tubular function by increasing RBF and GFR [92].

Reduced urine production and continuing urine drainage by venous and lymphatic systems together with tubular and renal pelvis dilatation result in a subsequent decline in renal pelvic pressure [48, 89, 93], which may be a compensatory mechanism to limit damaging increased intra-renal pressure [93].

### Initiation of proinflammatory cytokines

#### Cytokines in the stenotic PUJ

Transforming growth factor-beta (TGF-β) expression is noted in human stenotic PUJ compared to normal controls [94]. Furthermore, the smooth muscle regulators endothelin-1 (smooth muscle constrictor) and adrenomedullin (smooth muscle relaxant) have been shown to be increased and decreased, respectively, in stenotic PUJ disease [95].

Analysis of paediatric renal pelvis tissue proximal to the PUJO for cytokines that show altered renal expression in nephropathy demonstrates increased TGF-β and reduced macrophage inflammatory protein-1alpha (MIP-1α). In contrast, epidermal growth factor (EGF), monocyte chemotactic peptide 1, interferon-γ-inducible protein 10 and RANTES (regulated on activation normal T-cell expressed and secreted) mRNA expression are unchanged, suggesting that TGF-β and MIP-1α play important roles in the development of PUJO [88, 96].

#### Intra-renal cytokines

Increased intra-renal angiotensin II activates nuclear factor kappa B and ROCK (rho-associated coiled-coil-forming protein kinase), leading to cytokine release and interstitial macrophage infiltration and activation. Intra-renal selectins, integrins, intercellular-adhesion molecule 1, vascular cell adhesion molecule 1, interleukin 1, monocyte chemoattractant peptide 1, colony stimulating factor 1 and osteopontin expression are all involved in macrophage stimulation [11, 48, 88, 97]. Therefore, it appears that renal signals initiate and maintain the injurious inflammatory response to PUJO. Accordingly, both selectin and β2-integrin knockout mouse models show reduced macrophage infiltration into the obstructed kidney after UUO [43, 44].

### Inflammatory infiltrates

Activated macrophages infiltrate the renal interstitium, sustaining the inflammatory response by releasing cytokines, such as TGF-β1, tumour necrosis factor-alpha (TNF-α), and platelet-derived growth factor [11, 88].

### Profibrotic processes

Tubulointerstitial fibrosis is the final common pathway for many chronic kidney disorders, including obstructive uropathy, and is instigated by altered cytokine expression (Table 4). Activated resident interstitial myofibroblasts [98], expressing α-smooth muscle actin (boosts cell contractility) [99], aggregate, proliferate and produce extracellular matrix. Extracellular matrix consisting of collagens I, III and IV, fibronectin, laminin and proteoglycans accumulates due to increased synthesis and reduced degradation [74, 100, 101]. Myofibroblasts amplify fibrosis by producing cytokines, including TGF-β1 and TNF-α [11]. Parenchymal damage and renal dysfunction results, such that in children with PUJO the extent of fibrosis significantly correlates with differential renal function [102].

**Table 4:** Cytokines, growth factors, enzymes and adhesion molecules promoting or preventing tubulointerstitial fibrosis in ureteric obstruction

Molecules PROMOTING tubulointerstitial fibrosis in ureteric obstruction	Molecules PREVENTING tubulointerstitial fibrosis in ureteric obstruction
Angiotensin II	EGF
CTGF	MMP
ICAM-1	Nitric oxide
Integrins	VEGF
PAI-1	
PDGF	
TGF-β	
TIMP-1	

PAI-1, Plasminogen activator inhibitor 1; PDGF, platelet-derived growth factor

**Fig. 6:**
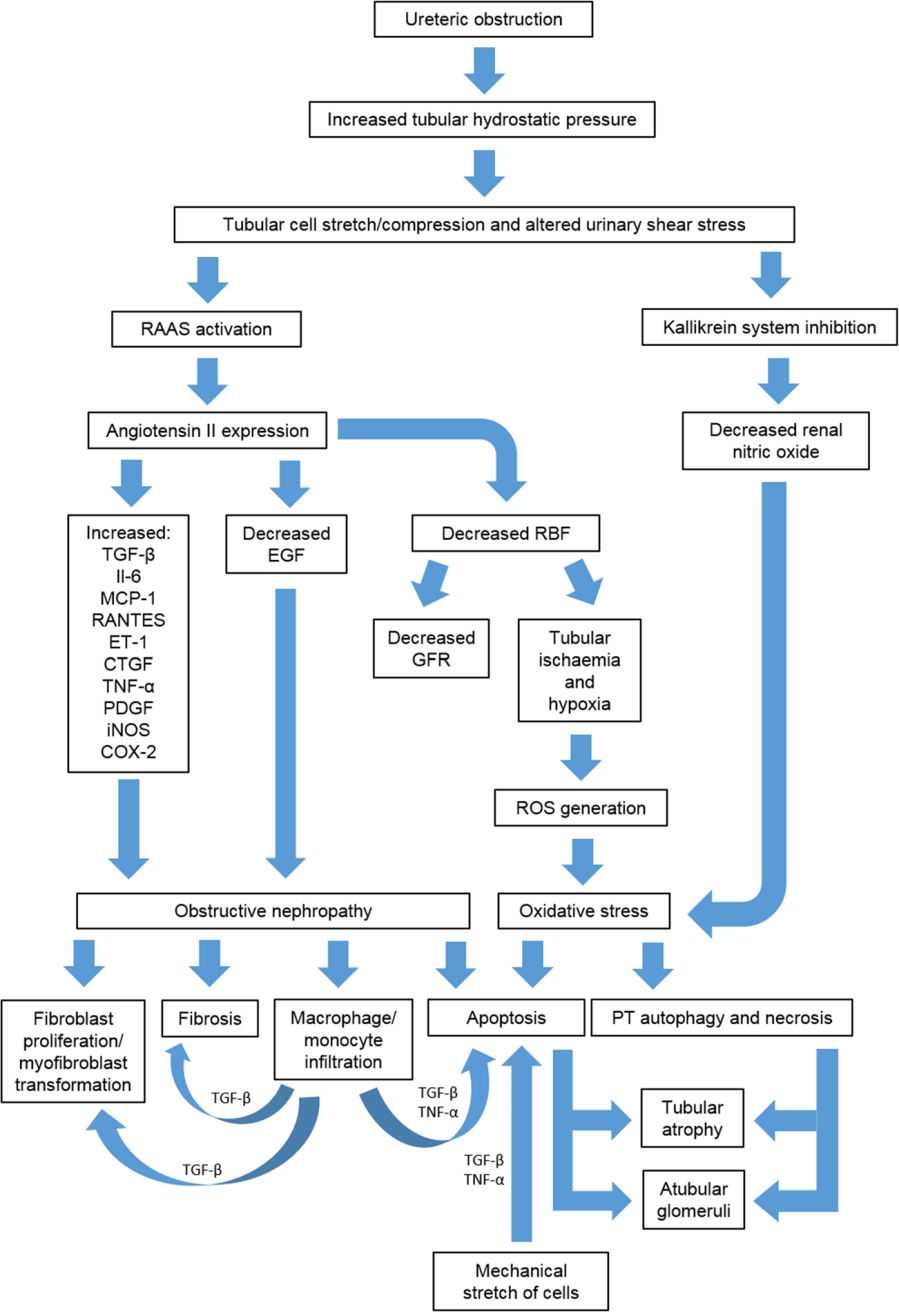
Major pathways involved in the development of obstructive nephropathy derived from animal and human studies. * ET-1* Endothelin 1,* iNOS* inducible nitric oxide synthase,* PT* proximal tubule,* RAAS* renin–angiotensin–aldosterone system,* RANTES* regulated on activation normal T-cell expressed and secreted,* RBF* renal blood flow,* ROS* reactive oxygen species. For other abbreviations, see footnotes to Tables 3 and 4

Angiotensin II upregulation is central to the pathogenesis of obstructive nephropathy (Fig. 6) [11, 41, 45, 64, 68, 83, 84, 91, 103–112]. Angiotensinogen murine knockout studies have demonstrated that angiotensin II expression is responsible for at least 50% of renal fibrosis in chronic neonatal UUO [104]. Acting predominantly via the AT1 receptor [45, 105, 113] it regulates cytokine production and stimulates reactive oxygen species (ROS) generation, which in turn propagates the proinflammatory, fibrogenic state [48, 104]. The generation of ROS also causes proximal tubular degeneration by apoptosis, autophagy and necrosis, with consequent destruction of the glomerulotubular junction, resulting in the formation of atubular glomeruli [41, 109].

**Fig. 7:**
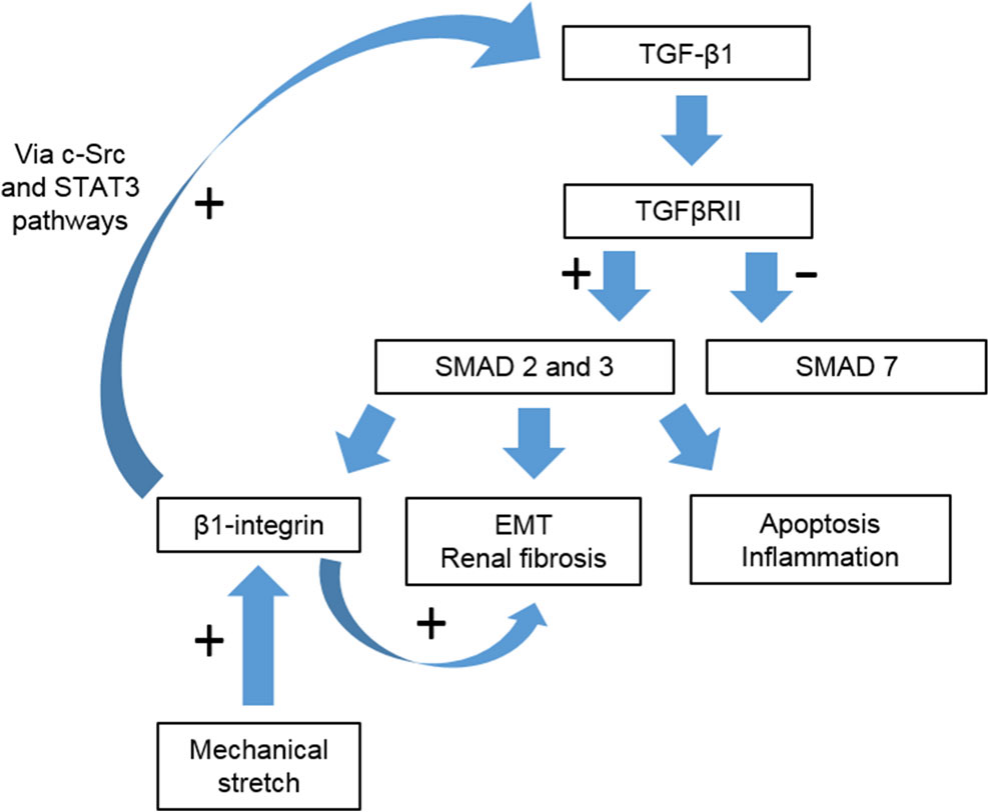
Transforming growth factor β1 (*TGF-β1*) signalling via the SMAD-dependent pathway. Unilateral ureteric obstruction induces increased TGF-β1 and TGF-β receptor II (*TGFβRII*) expression, upregulating SMAD 2 and 3 and downregulating SMAD 7 (inhibitory for SMAD 2 and 3). β1-integrin is upregulated by both SMAD signalling and mechanical stretch and contributes to a positive feedback loop regulating TGF-β1 expression via the c-SRC and STAT-3 pathways.* EMT* Epithelial mesenchymal transformation

TGF-β1 is a profibrotic cytokine and fibroblast chemo-attractant which plays a major role in fibrosis development via SMAD-dependent and -independent pathways (Fig. 7) [74–76, 114–118]. Renal TGF-β expression is increased in experimental UUO [83, 103, 105, 107, 119–121] and children with PUJO, being positively correlated with the histopathologic grade, radioisotope drainage half time (t1/2) and post-void washout and negatively correlated with pre-operative differential renal function [73, 122].

Nitric oxide is an endogenous vasodilator that protects against tubulointerstitial fibrosis and proximal tubular oxidant injury in obstructive nephropathy [79, 84, 123]. Animal models [111, 124, 125] and human studies of PUJO show altered endothelial NO synthase (eNOS) and inducible NO synthase (iNOS) expression/activity together with reduced NO production. Lower eNOS expression/activity is associated with worse creatinine clearance, reduced differential renal function [90, 126] and increased fibrosis [90, 126], oxidant injury and apoptosis [67, 79].

### Antifibrotic processes

Renal cyclooxygenase 2 (COX-2) expression and prostaglandin production in experimental UUO is increased [69] and may be a protective response. COX-2 inhibition worsens obstructive nephropathy, while prostacyclin analogue (ONO-1301) supplementation alleviates UUO-induced fibrosis [127].

### Cellular apoptosis

Apoptosis affects podocytes and endothelial and epithelial cells within the kidney, leading to loss of glomeruli, peritubular capillaries and tubules [11]. Tubular cell mechanical stretch is a potent stimulator of apoptosis [91, 128] that is mediated via TGF-β1 and TNF-α [68, 110] released from tubular cells and infiltrating macrophages [88]. Other proapoptotic factors increased after UUO include Fas-L [45], p53, caspases and ceramide [11].

Downregulation of anti-apoptotic factors, including EGF, eNOS, NO, vascular endothelial growth factor, heat shock protein 70 and Wilms tumour-1, compounds the renal injury [11, 67, 88, 128, 129].

### Tubular function impairment

Ureteric obstruction leads to reduced renal expression of the V2 (vasopressin) receptor [130], renal sodium and urea transporters [131–133] and aquaporins [134–136]. Aquaporins are a family of transmembrane proteins normally expressed by mammalian kidney [137] and urothelium [138, 139] that mediate water movement across the cell membrane along an osmotic gradient [140]. Reduced renal aquaporin expression in experimental UUO is noted within 24 h of complete obstruction [134]. Similarly, renal aquaporins are downregulated in children undergoing pyeloplasty, and in both human and animal models this reduction is associated with polyuria and reduced concentrating ability following relief of obstruction [141–143].

## Genetic mechanistic clues in PUJO

Phenotypes similar to PUJO have been noted in numerous transgenic mouse models. Many genes involved in ureteric smooth muscle proliferation and differentiation are implicated, supporting a primary myogenic aetiology. Importantly, one of these genes has been implicated in human disease (Table 2).

Mutations in *TBX18*, the gene coding for T Box protein 18, have been reported in association with congenital anomalies of the kidney and urinary tract (CAKUT). In particular, a heterozygous *TBX18* truncating mutation (c.1010delG) showing autosomal dominant inheritance has been described across four generations of a family with CAKUT, and predominantly PUJO [56]. The transcription factor TBX18 is necessary for normal smooth muscle cell proliferation, differentiation and localisation around the developing urothelial stalk [24]. TBX18 also directs epithelial proliferation and when absent leads to an abnormally short ureteric bud [28].

In the majority of patients, however, PUJO is a polygenic disorder without an obviously inherited genetic component [11].

## Potential therapeutic molecular targets in PUJO

Human and animal studies have highlighted a number of potential therapeutic targets that could be manipulated to alleviate the nephropathy sustained secondary to PUJO. Several drugs targeting these pathways have been assessed in rodent UUO models as described below, however, to our knowledge none of these therapies have been trialed in childhood human PUJO.

### Angiotensin-converting enzyme and AT1 receptor inhibitors

In adult rodent UUO models angiotensin-converting enzyme (ACE) inhibitors and AT1 receptor inhibitors given prophylactically (for the duration of obstruction) are beneficial in alleviating nephropathy. Specifically, they reduce TGF-β [121, 144] and TNF-α [106] expression, as well as macrophage infiltration and tubulointerstitial fibrosis [84, 105, 145]. Additionally, AT1 receptor inhibitors improve tubular function by improving RBF and GFR and attenuating the reduction in sodium transporter and aquaporin 2 (AQP2) expression, thus reducing polyuria and natriuresis [92, 112].

ACE inhibitors reduce both AT1 and AT2 receptor stimulation [146] and indirectly increase NO levels via bradykinin generation [84]. This may explain why they confer additional benefits, particularly anti-inflammatory, compared to AT1 receptor inhibitors [97]. Unfortunately, inhibition of angiotensin during either the period of nephrogenesis (first 10 days after UUO) or renal maturation (second 10 days after UUO) in neonatal partial UUO exacerbates renal injury in both the obstructed and contralateral kidney [147, 148]. Such studies highlight the importance of these pathways in normal kidney development and maturation.

However, it is important to remember that ACE inhibitors and AT1 receptor inhibitors are frequently used in children with chronic kidney disease, in whom they significantly reduce proteinuria [149] despite not significantly alleviating the natural decline in excretory function [150, 151]. They are largely well tolerated, with no apparent effect on growth and development and a low incidence of side effects such as hyperkalaemia, hypotension and renal injury [149].

### Hydroxymethylglutaryl-CoA reductase inhibitors (statins)

Statins ameliorate nephropathy when administered prophylactically in adult and neonatal rodent UUO models by reducing renal cytokine production (TGF-β, TNF-α), macrophage infiltration, oxidative stress, apoptosis and tubulointerstitial fibrosis [85, 152, 153]. These pleiotropic effects are achieved through decreased Ras/ERK/Akt signalling [154] and increased NO bioavailability [155]. Importantly, statins remain beneficial in neonatal rodent UUO where an improvement in tubular dilatation and glomerular number and size are also seen [67, 79, 85]. Functionally, in UUO models, statins improve GFR and microalbuminuria [156] and increase urinary concentrating ability via boosting AQP2 expression [157].

Statins are commonly used and usually well tolerated in adults. Side effects of treatment include hepatic dysfunction, diabetes mellitus, benign proteinuria, peripheral neuropathy, myalgia and rhabdomyolysis [158]. A 10-year follow-up study of children (≥8 years) treated with statins for familial hypercholesterolaemia demonstrated that few discontinue therapy due to side effects and that there were no serious adverse reactions [159]. In that same study, growth, puberty and educational parameters were also unaffected compared to controls [159].

### TGF-β modulation

Prophylactic TGF-β receptor inhibition is renoprotective in adult rodent UUO models, reducing apoptosis, macrophage infiltration, fibrosis, proximal tubular atrophy and atubular glomeruli formation [117, 160]. Similarly, anti-TGF-β antibody treatment increases NOS expression while reducing apoptosis and fibrosis [110]. Conversely, prophylactic TGF-β receptor inhibition in neonatal mouse UUO causes widespread renal necrosis, exacerbating the injury in the obstructed kidney and highlighting the differing responses to signalling cascades during renal development [117].

Anti-TGF-β antibody treatment (GC1008) has been trialled in human oncological disease and was generally well tolerated. However, side effects included gingivitis, fatigue and skin rashes, including keratoacanthoma and squamous cell carcinoma development (melanoma patients only). GC1008 treatment has not progressed beyond phase II clinical trials as drug development was discontinued by the manufacturer [161].

### COX-2 inhibition

In adult rodent bilateral ureteric obstruction COX-2 inhibition alleviates AQP2 and sodium transporter downregulation and improves post-obstructive polyuria, which would appear to be beneficial [69]. Conversely, other studies have demonstrated that both genetic COX-2 knockout and prophylactic COX-2 inhibition in adult rodent UUO models increase tubular injury, apoptosis and fibrosis, thereby negating potential use in the clinical setting [70, 162].

Chronic celecoxib (COX-2 inhibitor) use in children demonstrates a similar frequency of adverse events to non-selective non-steroidal anti-inflammatory drugs, which are most frequently gastrointestinal side effects [163].

### Other potential therapeutic options

Other potential therapeutic pathways include those that are able to increase the vasoactive molecule NO, as this has been shown to reduce tubulointerstitial fibrosis in adult rodent UUO models [84]. Although both ACE inhibitors and statins increase NO bioavailability, this is an indirect effect at the expense of drug-related side effects.

Dietary nitrate supplementation is a novel therapeutic option which directly targets the NO pathway, increasing NO generation via nitrite production. Nitrite also has cytoprotective effects independent of NO by influencing mitochondrial function [164], and when administered during rodent ischaemia reperfusion studies reduces renal injury [165].

Despite former concerns associating nitrates with methaemoglobinaemia and carcinogenesis, the nitrate–nitrite–NO pathway is increasingly implicated in a protective role in humans [166]. Further investigation of dietary nitrate supplementation as a potential therapy in obstructive nephropathy is warranted.

## Urinary biomarkers

Identifying early urinary biomarkers in PUJO may be beneficial for the diagnosis, management and prognosis of this condition. Such biomarkers would enable timely detection of children with ‘damaging’ hydronephrosis who require surgery to protect renal function, while avoiding surgery in those with ‘safe’ hydronephrosis.

### Urinary biomarkers in animal studies

There is little data on urinary biomarkers from animal studies. Proteomics using a rat UUO model demonstrated increased urinary and renal levels of alpha-actinin-1 and moesin at 1 week which corresponded with histological evidence of tubular injury. Following 3 weeks of UUO urine and renal levels of vimentin, annexin A1 and clusterin were significantly elevated, corresponding with substantial renal interstitial fibrosis [167].

### Urinary biomarkers in human studies

Many urinary cytokines, growth factors, chemokines, tubular enzymes and tubular transport proteins have been investigated in children undergoing pyeloplasty for PUJO. Studies with conservatively managed PUJO as a comparator are most useful to identify biomarkers able to aid selection of patients for surgery. Potential urinary biomarker proteins measured in bladder urine samples are presented in Table 5.

**Table 5:** Urinary proteins from studies in children with pelvi-ureteric junction obstruction

Urinary protein (corrected for creatinine)^a^	Primary measured group	Comparators	Bladder urine protein level	Sensitivity/specificity/accuracy^b^	Post-operative bladder urine (compared to pre-operative)	Ref
ALP	Pyeloplasty	CMP	Increased pre-operative	Se 91.4%/ Sp 100%/ Ac 94%	Decreased 12 months post-operative	[168]
Angiotensinogen	Pyeloplasty	Healthy control CMP	Increased pre-operative	Se 93.3%^c^/ Sp 60%^c^		[169]
B2-microglobulin	PUJO*	Healthy control	Increased		Decreased 42 months post-operative	[170]
B2-microglobulin	Pyeloplasty	Healthy control	No change			[171]
Ca19-9	Pyeloplasty	Healthy control CMP	Increased pre-operative	Se 76%^d^/Sp 85%^d^	Decreased 3 months post-operative	[172]
Ca19-9	Pyeloplasty	Healthy control Hydrocoele/renal cyst	Increased pre-operative	Se 100%^e^/ Sp 82.6%^e^	Decreased 3 months post-operative	[173]
CyC	Pyeloplasty	Healthy control	No change			[171]
EGF	PUJO*	Healthy control	Decreased (obstructed group only)		No change	[170]
EGF	Pyeloplasty	Healthy control	Decreased pre-operative		Increased	[174]
EGF	Pyeloplasty	Healthy control	Increased pre-operative	Se 70.4%/Sp 69.2%	Decreased 3 months and 1 year post-operative	[175]
EGF	Pyeloplasty	Healthy control	No change			[176]
ET-1	Pyeloplasty	Healthy control VUR Renal stones	Increased pre-operative	Se 74.3%/Sp 90%/ Ac 81.5%	Decreased 12 months post-operative	[177]
γGT	Pyeloplasty	CMP	Increased pre-operative	Se 62.9%/Sp 100%/Ac 74%	Decreased 12 months post-operative	[168]
HO-1	Pyeloplasty	Healthy control CMP	Increased pre-operative	Se 72.2%^c^/Sp 78.1%^c^	Decreased 1 month post-operative	[178]
IP-10	Pyeloplasty	Healthy control	No change			[175]
KIM-1	Pyeloplasty	Healthy control CMP	Increased pre-operative	Se 100%^c^/Sp 71.4%^c^		[179]
MCP-1	Pyeloplasty	Healthy control	Increased pre-operative	Se 77.8%/Sp 69.2%	Decreased 3 months and 1 year post-operative	[175]
MCP-1	PUJO*	Healthy control	Increased		Decreased 42 months post-operative	[170]
MCP-1	Pyeloplasty	Healthy control	Increased pre-operative			[174]
MCP-1	Pyeloplasty	Healthy control CMP	Increased pre-operative	Se 100%^c^/Sp 0%^c^	Remains high 3 months post-operative	[180]
MIP-1α	Pyeloplasty	Healthy control	Decreased pre-operative		Increased 1 year post-operative	[175]
NAG	Pyeloplasty	CMP	Increased pre-operative	Se 97.1%/Sp 80%/Ac 92%	Decreased 12 months post-operative	[168]
NGAL	Pyeloplasty	Healthy control	No change			[171]
NGAL	Pyeloplasty	Healthy control	Increased pre-operative			[181]
NGAL	Pyeloplasty	Healthy control CMP	Increased pre-operative	Se 100%^c^/Sp 28.6%^c^	Decreased 3 months post-operative	[179]
OPN	Pyeloplasty	Healthy control	No change			[171]
OPN	Pyeloplasty	Healthy control CMP	Increased pre-operative	Se 98.5%^c^/Sp 10.5%^c^	Remains high 3 months post-operative	[180]
RANTES	Pyeloplasty	Healthy control	No change			[175]
TGF-β	Pyeloplasty	Healthy control	Increased pre-operative	Se 100%/Sp 80%/Ac 90.8%	Decreased 1 year post-operative	[176]
TGF-β	Pyeloplasty	CMP	Increased pre-operative	Se 82%/Sp 86%		[182]

Generally, the primary group measured is children undergoing pyeloplasty; these children are then compared to healthy controls and/or conservatively managed children with PUJO (CMP). The exception in the studies listed in the table is labelled PUJO*, which includes children with conservatively managed PUJO split into ‘functional’ (t1/2 of renogram < 0 min) and ‘obstructed’ (t1/2 of renogram > 20 min). In these studies voided urine from children undergoing pyeloplasty was only obtained 42 months post-operative

^a^ALP, Alkaline phosphatase; Ca19-9, carbohydrate antigen 19–9; CyC, cystatin-C; HO-1, heme oxygenase-1; γGT, gamma-glutamyl transferase; IP-10, interferon-γ-inducible protein 10; KIM-1, kidney injury molecule-1; MIP-1α, macrophage inflammatory protein-1α; NAG,* N*-acetyl-beta-D-glucosaminidase; NGAL, neutrophil gelatinase-associated lipocali; OPN, osteopontinn, RANTES, regulated on activation normal T-cell expressed and secreted

^b^Where applicable sensitivity (Se), specificity (Sp) and accuracy (Ac) of the test at best threshold value from receiver operating characteristic curve analysis is presented

^c^To detect differential renal function (DRF) of <40% out of all hydronephrosis cases

^d^To detect pyeloplasty cases out of all hydronephrosis cases

^e^To detect pyeloplasty cases out of all cases

Finding a suitable biomarker test with high sensitivity, specificity and predictive value is challenging [88], not least because these markers are excreted in health as well as disease, show significant intra- and inter-patient variation and may be affected by patient age, the presence of urinary tract infection and other renal disorders [174, 183].

A recent systematic review of urinary and serum biomarkers included 14 studies which reported data on 380 surgically managed PUJO patients, 174 conservatively managed patients and 213 controls [184]. This review reported a wide-range of sometimes conflicting results, and the authors were unable to draw any firm conclusions, attributing this to differences in study design with heterogeneous age groups, various or absent control groups and often short durations of follow-up [184].

More successfully, proteomics of neonatal urine has identified a panel of 51 peptides which distinguish obstruction severity. When implemented in a prospective blinded study it had an accuracy of 94% to predict future need for surgery in newborns with PUJO [185]. However, beyond 1 year of age the sensitivity and specificity of this proteome profile diminished significantly [186].

A single biomarker able to guide selection of patients for pyeloplasty has not yet been identified, indicating a panel of biomarkers may be necessary to achieve this.

## Conclusions

Managing children with asymptomatic intrinsic PUJO is a significant challenge for clinicians. Animal and human studies have expanded our understanding of the molecular mechanisms involved in the aetiology of obstruction and in particular the progression of the renal insult. Upregulation of the RAAS and TGF-β expression are fundamental to renal injury, which is attenuated in animal models by therapeutic inhibition of these pathways. Much, however, remains to be learned in order to identify molecular markers and targets useful in the day-to-day diagnosis and management of this condition.

## Future perspectives and unanswered questions in PUJO

- What is the underlying aetiology of intrinsic congenital PUJO? Does this explain the variable outcome of PUJO and can this be targeted therapeutically?
- Does individual ability to relieve intra-renal pressure determine disease progression?
- Are therapies tested in animals applicable in children to limit renal injury?
- Can urinary biomarkers improve early identification and thus outcome of children requiring pyeloplasty?
